# Association of Women’s Health Literacy and Work Productivity among Japanese Workers: A Web-based, Nationwide Survey

**DOI:** 10.31662/jmaj.2019-0068

**Published:** 2020-07-13

**Authors:** Yuko Imamura, Kazumi Kubota, Naho Morisaki, Shu Suzuki, Mariko Oyamada, Yutaka Osuga

**Affiliations:** 1 Health and Global Policy Institute, Tokyo, Japan; 2 Department of Biostatistics, Graduate School of Medicine, Yokohama City University, Kanagawa, Japan; 3 Division of Life-course Epidemiology, Department of Social Medicine, National Center for Child Health and Development, Tokyo, Japan; 4 Department of Community Nursing, Graduate School of Medicine, The University of Tokyo, Tokyo, Japan; 5 Department of Obstetrics and Gynecology, Graduate School of Medicine, The University of Tokyo, Tokyo, Japan

**Keywords:** Women’s Health Literacy, Work Productivity, Japanese Workers, A Web-Based Nationwide Survey

## Abstract

**Introduction:**

This study examined the relationship between health literacy (HL), women’s health, and work productivity (i.e., absenteeism or presenteeism) among female workers in Japan.

**Methods:**

In February 2018, a web-based, nationwide survey was conducted among registered survey company monitors. The questionnaire included women’s HL, absenteeism, presenteeism, health behaviors for menstrual abnormalities and premenstrual syndrome (PMS), and demographic information. Overall, 2,596 monitors were randomly invited, and the survey included the first 2,000 respondents (average age = 35.8 years, SD = 8.1). An analysis of covariance (ANCOVA) was conducted to compare adjusted work productivity between two groups: the low-HL group and the high-HL group. The results were adjusted for age, education, employment status, number of children, and the presence of underlying gynecological diseases. Logistic regression analyses were performed to determine any differences in health behaviors for menstrual abnormalities or PMS between the two groups. The results were adjusted for age, education level, number of children, and employment status.

**Results:**

The ANCOVA showed that the high-HL group had significantly less presenteeism and better performance when experiencing PMS (p < 0.001 and p < 0.013, respectively) compared to the low-HL group after adjusting for covariates. However, the results showed no significant differences in absenteeism between the two groups. Logistic regression showed that the high-HL group had a significantly higher odds ratio (OR) than the low-HL group in terms of health behaviors for menstrual abnormalities or PMS (OR 2.82 and 1.86, respectively) after adjusting for covariates.

**Conclusions:**

Women’s HL may contribute to decreased presenteeism and better health behaviors regarding the use of medicine or medical services.

## Introduction

Japan’s labor force is projected to decrease dramatically due to the declining birthrate and the aging of the Japanese population ^(1)^. Most Japanese workers are male; thus, the government implemented a policy promoting female participation in the labor force ^(2)^. In addition, as the labor force shrinks, it will be more important than ever to increase individual work performance to maintain economic growth.

Health behaviors influence work performance ^(3)^; positive health behaviors lead to preventive health measures and investments in health, which can improve and maintain workers’ health status and increase work performance ^(4), (5)^. Recent studies show that health literacy (HL), defined as “the cognitive and social skills which determine the motivation and ability of individuals to gain access to, understand and use information in ways which promote and maintain good health,” ^(6)^ is necessary to improve health behaviors ^(7)^. Low-HL levels can lead to excessive anxiety over inaccurate health information ^(8)^.

Although the connection between HL and health behaviors, and the connection between good health behaviors and increased work performance have been investigated, HL’s influence on labor productivity has not been studied extensively. On the other hand, the Japanese government has established a policy of promoting women’s active participation in the workforce. Key Policies for Accelerating the Empowerment of Women 2019 also emphasizes the need to strengthen lifetime health support of women ^(9)^. In this context, HL may be an especially important issue for women. Up to 25% of reproductive-aged women experience premenstrual syndrome (PMS); how they control PMS may differ, based on their HL level, and influence their work performance ^(10)^. Hence, it is important to identify changes in physical conditions unique to women, such as PMS, and to encourage them to gain appropriate literacy to live and work in a healthy manner.

Therefore, we examined the relationship between HL on women's health and labor productivity among female workers in Japan. The ages and regional profiles of the survey cohort matched Japan’s national demographic profile.

## Materials and Methods

### 1. Participants

An internet-based survey was conducted among registered monitors of Cross Marketing Inc. The survey included questions about women’s HL and work performance. The survey period was from February 2, 2018, to February 8, 2018. Cross Marketing Inc. has approximately 4.2 million registered monitors across Japan. This population has a balanced age distribution, with 20.0% of the monitors in their 30s, 24.0% in their 40s, and 19.0% in their 50s. As of May 2018, the main occupations among Cross Marketing Inc. monitors included company employees (i.e., general staff) (26.0%), part-time workers (14.0%), and full-time homemakers (13.0%). To exclude multiple responses from the same monitor, we: 1) identified duplicates by comparing each monitor’s gender, birthdate, and postal code, recorded at the time of registration and 2) compared IP addresses to ensure that each response came from a unique IP address. Survey invitations were sent, via e-mail, to 2,596 women between the ages of 18 and 49, who were randomly selected from the overall monitor population. The survey cohort’s ages and regional profiles matched Japan’s national demographic profile of female workers ^(11)^. The survey included the first 2,000 monitors who responded to the invitation (average age = 35.8 years, SD = 8.1). Before starting the survey, all participants consented to publish the information. The survey was conducted with the approval of the Ethics Review Committee of the Health Outcome Research Institute, Japan (approval code is 2018-001).

### 2. Survey items

#### 1) Measurements of HL on women’s health

The HL levels were measured by the Health Literacy Scale for Women of Reproductive Age ^(12)^ (hereafter referred to as the Health Literacy Scale), which was developed for the prevention and early detection of women’s health issues among working women in Japan. The Health Literacy Scale consists of four categories: women’s choices and practices related to health information (e.g., “There are specific activities that I regularly do to maintain my health”; α = 0.92), self-care during menstruation (e.g., “I understand my menstrual cycle”; α = 0.84), knowledge of the female body” (e.g., “I know about the mechanisms of pregnancy”; α = 0.89), and sexual health discussions with partners (e.g. “I can discuss preventing sexually transmitted diseases (STDs) with my partner when necessary”; α = 0.85). The scale contains 21 items. Respondents were asked to choose appropriate answers from a 4-point Likert scale, ranging from 1 = “Agree” to 4 = “Disagree” for each item. The HL Scale’s reliability and validity were confirmed by Kawata et al　^(12)^.

#### 2) Measurements of work productivity

Work productivity was measured by the validated Japanese version of the World Health Organization’s Health and Work Performance Questionnaire (HPQ) ^(13), (14)^. The HPQ measures both productivity loss due to absence (i.e., absenteeism) and productivity loss due to poor physical or mental functioning when the person continues to report for work (i.e., presenteeism). Higher values for absolute absenteeism indicate lower work productivity levels (more sick leave over a four-week period). Higher values of absolute presenteeism also indicate lower work productivity levels (compared to the best possible work performance a person could have on the job). The questionnaire includes questions regarding work performance over the previous four weeks. The HPQ’s reliability and validity were confirmed by Miyaki et al ^(14)^.

In this study, absolute absenteeism and presenteeism were calculated based on the HPQ scoring guidelines. The calculation method for absolute absenteeism was as follows: Absolute Absenteeism = 4 × (score for “How many hours does your employer expect you to work in a typical week?”) - (score for “How many hours have you worked in the previous four weeks?”). The calculation method for absolute presenteeism was as follows: 10 × (self-rated overall work performance during the past four weeks on a 10-point Likert scale ranging from “worst possible work performance a person could have on the job” to “best work performance”).

#### 3) Measurements of menstrual abnormalities, PMS, and health behaviors

The respondents were asked whether they had ever experienced or were currently experiencing menstrual abnormalities. In this survey, the following symptoms were considered menstrual abnormalities, based on guidelines reported by The Japan Society of Obstetrics and Gynecology ^(15)^: irregular and unpredictable menstrual cycles; short cycles (menstruation reoccurring within 24 days); long cycles (menstruation reoccurring after 39 days); missed periods for several months; minimal bleeding during menstruation; excessive bleeding during menstruation; abnormally short periods (two days or fewer); abnormally long periods (more than eight days); severe symptoms during menstruation (including severe lower abdominal pain, lower back pain, abdominal bloating, nausea, headache, fatigue, weakness, loss of appetite, irritability, diarrhea, or depression); and abnormal bleeding.

Similarly, the survey also asked women if they had ever experienced or were currently experiencing PMS. PMS was defined as the presence of the following symptoms, based on the guidelines published by the Japan Society of Obstetrics and Gynecology ^(15)^: physical or mental discomfort before menstruation, including irritability, impatience, being emotional or feeling sad, weariness/fatigue, daytime drowsiness, sleeplessness, breast stiffness or pain, swelling, headaches, neck or shoulder stiffness, nausea, cold fingers or toes, rashes, dizziness, increased appetite, rough skin, or weight gain.

In this study, we also used a set of original questions about how PMS and menopausal symptoms or disorders influence work, which we developed from the presenteeism items on the HPQ, including the following: “How much does your work performance change due to PMS or menstrual symptoms? Please rate your performance when experiencing such symptoms, with 10 being your usual performance. If you feel your performance fluctuates, please give your average value.” These scores were multiplied by 10, and the values ranged from 0 (the worst performance) to 100 (the best performance).

We also included several questions about health behaviors related to menstrual abnormalities or PMS. Women were considered to have good health behavior if they reported taking medication (either prescription or over-the-counter medications) or visiting a physician (e.g., obstetrician/gynecologist or general medicine physician) due to menstrual abnormalities or PMS.

#### 4) Demographics

The survey collected demographic information, including age, number of children (zero, one, two, or three or more), education level (junior college or less or four-year college or beyond), employment status (full-time regular or temporary staff), and the presence of underlying gynecological illnesses. For this survey, underlying gynecological illnesses were defined as STDs (HIV, syphilis, chlamydia, etc.), cervical cancer, uterine cancer, ovarian cancer, breast cancer, endometriosis, uterine myoma, polycystic ovary syndrome, PMS, menstrual symptoms, and menopausal disorders.

### 3. Analysis methodology

In this study, HL was our exposure of interest. Our primary outcome of interest was work productivity, which was measured by absenteeism and presenteeism scales. Our secondary outcome of interest was the practice of health behaviors.

Thus, we grouped respondents into one of two groups, depending on their HL level, which was based on the results from the previously described scales, and conducted all analyses using these two groups. Respondents reporting HL scores equal to, or greater than, the median score were considered to have high-HL, while those with scores lower than the median score were considered to have low-HL.

We first used either the analysis of variance or Chi-square tests to compare and investigate demographics in the high-HL and low-HL groups.

Then, analysis of covariance (ANCOVA) were performed. High or low-HL was the independent variable, and work performance was the dependent variable. The ANCOVA was first adjusted for demographic characteristics, such as age, education level, employment status, number of children, and the presence of underlying gynecological diseases (Model 1). Health behaviors for PMS was then also adjusted in Model 2.

Furthermore, we conducted a logistic regression, with HL as the independent variable and the practice of health behaviors for menstrual abnormalities or PMS (yes or no) as the dependent variable. For this analysis, only respondents who answered “I currently have symptoms” or “I had symptoms in the past” were included in the logistic regression analysis. The logistic regression results were adjusted for age, education level, number of children, and employment status. IBM SPSS version 22.0 was used for statistical analyses.

## Results

### 1. Demographics

Table 1 summarizes demographic variables, work performance, and health behaviors pertaining to menstrual abnormalities or PMS among the two HL groups (i.e., high-HL and low-HL). The high-HL group had significantly higher presenteeism scores than the low-HL group. The results also showed a significant difference between the two groups in terms of the number of children, presence of underlying gynecological diseases, and health behaviors for menstrual abnormalities or PMS. For PMS, 29.8% of the high-HL respondents demonstrated health behaviors, while only 17.1% of the low-HL respondents demonstrated health behaviors. However, there were no significant differences observed in age, employment status, absenteeism scores, and work performance between the two groups while experiencing PMS.

**Table 1.** Demographic Characteristics (All Women, N = 2,000)^1)^.

	High Health Literacy (Score ≧ 56)					Low Health Literacy (Score ≦ 55)				Statistical test (degrees of freedom), test statistic	p-value
	n	(%)	Mean	SD^2)^		n	(%)	Mean	SD^2)^		
Age	1,015		35.9	(8.9)		985		35.8	(14.0)	F (1, 1998) = 0.137	0.712
Age groups										χ^2^ (2) = 0.538	0.764
under 29	298	(29.4)				303	(30.8)				
30-39	331	(32.6)				310	(31.5)				
40-49	386	(38.0)				372	(37.8)				
Number of children											
None	792	(72.1)				802	(81.4)				
One	154	(15.2)				90	(9.1)			χ^2^ (3) = 25.791	< 0.001
Two	92	(9.1)				70	(7.1)				
Three or more	37	(3.6)				23	(2.3)				
Education											
Junior college or under	454	(44.7)				544	(55.2)			χ2 (1) = 22.042	< 0.001
Four-year college or over	561	(55.3)				441	(44.8)				
Employment status											
Full-time regular staff	782	(77.0)				745	(75.6)			χ^2^ (1) = 0.550	0.458
Full-time temporary staff	233	(23.0)				240	(24.4)				
Presence of underlying gynecological diseases											
Yes	312	(30.7)				169	(17.2)			χ^2^ (1) = 22.042	< 0.001
No	703	(69.3)				816	(82.8)				
Work performance											
Absenteeism (HPQ score)	1,015		21.5	(54.2)		985		20.5	(55.3)	F (1,1998) = 0.164	0.685
Presenteeism (HPQ score)	1,015		65.5	(18.5)		985		57.1	(17.5)	F (1,1998) = 86.240	< 0.001
Presenteeism while experiencing PMS	1,015		59.7	(21.2)		985		58.1	(21.7)	F (1,1998) = 2.864	0.091
Health behaviors for menstrual abnormalities											
Yes	354	(34.9)				187	(19.0)			χ^2^ (1) = 70.407	< 0.001
No	178	(17.5)				264	(26.8)				
No reported symptoms	483	(47.6)				534	(54.2)				
Health behaviors for PMS											
Yes	302	(29.8)				168	(17.1)			χ^2^ (1) = 49.368	< 0.001
No	408	(40.2)				423	(42.9)				
No reported symptoms	305	(30.0)				394	(40.0)				

1) p-values were calculated as noted using analysis of variance (ANOVA) or Chi-square tests 2) SD: Standard Deviation 3) HPQ: Health and Work Performance Questionnaire 4) PMS: premenstrual syndrome

### 2. Association between HL and work performance

Table 2 compares work performance between the high-HL and low-HL groups. After adjusting for age, education level, employment status, number of children, the presence of underlying gynecological diseases, and health behaviors for PMS, the high-HL group had significantly higher presenteeism scores, and better performance, when experiencing PMS (p < 0.001 and p < 0.013, respectively) than the low-HL group. However, the results showed no significant difference in absenteeism scores between the two groups.

**Table 2.** Association between Health Literacy Levels (High or Low) and Work Performance (All Women, N=2,000)^1)^.

	Absenteeism (HPQ score)					Presenteeism (HPQ score)					Presenteeism while experiencing PMS^3)^			
Health Literacy (HL)	n	Adjusted mean	SE^2)^	95%CI^2)^		n	Adjusted mean	SE^2)^	95%CI^2)^		n	Adjusted mean	SE^2)^	95%CI^2)^
Model 1^4)^														
High HL (Score ≧56)	1,015	21.3	(1.7)	(18.33-25.01)		1,015	64.6	(0.6)	(63.52-65.73)		1,015	60.6	(0.7)	(58.69-61.37)
Low HL (Score ≦55)	985	20.3	(1.7)	(16.90-23.74)		985	57.0	(0.6)	(55.85-58.10)		985	57.8	(0.7)	(56.43-59.16)
		p = 0.576					p < 0.001					p = 0.023		
Model 2^5)^														
High HL (Score ≧56)	710	18.9	(2.0)	(15.01-22.77)		710	63.9	(0.7)	(62.54-65.24)		710	57.6	(0.8)	(56.02-59.20)
Low HL (Score ≦55)	591	21.0	(2.1)	(16.82-25.25)		591	55.6	(0.8)	(54.01-57.04)		591	54.6	(0.9)	(52.83-56.32)
		p = 0.474					p < 0.001					p = 0.013		

1) All analyses were conducted using analysis of covariance (ANCOVA). 2) SE = standard error; CI = confidence interval. 3) PMS: Respondents were asked to choose either 1) “I currently have symptoms,” 2) “I had symptoms in the past,” or 3) "Never." Women who chose 1) or 2) were included in this analysis (n = 1,301). 4) Adjusted for age, education level, employment status, number of children, and the presence of underlying gynecological diseases. 5) Adjusted for Health behaviors for PMS in addition to variables adjusted in Model 1.

### 3. Association between HL and health behaviors for menstrual abnormalities or PMS^1)^.

Table 3 displays the logistic regression analysis results. After adjusting for age, education level, number of children, and employment status, the high-HL group had a significantly higher OR than the low-HL group for health behaviors pertaining to menstrual abnormalities or PMS (OR 2.82 (95% CI 2.16–3.67) and OR 1.86 (95% CI 1.47–2.36), respectively).

**Table 3.** Association between Health Literacy Levels (High or Low) and Coping Behaviors for Menstrual Abnormalities or PMS (All Women).

	Health behaviors for menstrual abnormalities^3)^					Health behaviors for PMS^4)^			
	n	OR^2)^	95% CI^2)^	p-value		n	OR^2)^	95% CI^2)^	p-value
Health Literacy (HL)								
High HL (Score ≧56)	532	2.82	(2.16-3.67)	< 0.001		710	1.86	(1.47-2.36)	< 0.001
Low HL (Score ≦55)	451	1.00		< 0.001		591	1.00		< 0.001

1) All analyses were conducted using binary logistic regression adjusting for age. 2) OR = odds ratio; CI = confidence interval. 3) Menstrual abnormalities: Respondents were asked to choose either 1) “I currently have symptoms,” 2) “I had symptoms in the past,” or 3) “Never.” Women who chose 1) or 2) were included in this analysis (n = 983). 4) PMS: Respondents were asked to choose either 1) “I currently have symptoms,” 2) “I had symptoms in the past,” or 3) “Never.” Women who chose 1) or 2) were included in this analysis (n = 1,301).

## Discussion

In this nationwide survey among Japanese female workers, we observed that women with high-HL had better work performance while experiencing PMS and were more likely to demonstrate health behaviors pertaining to PMS than those with low-HL. These associations remained even after adjusting for women’s background characteristics related to HL, such as education level, number of children, and the presence of underlying gynecological diseases.

### 1. Association between women’s background characteristics and HL

We found that patients’ medical experiences and knowledge affects their understanding of health information ^(16)^ since women with higher education levels, those with underlying gynecological diseases, and those with children had higher HL. Several previous studies have revealed a positive association between higher education levels and HL ^(17), (18)^. Mancuso indicated that consultation and treatment experiences related to underlying diseases may enhance knowledge levels and self-care capabilities ^(16)^. No previous study has reported an association between women’s experiences with pregnancy and childbirth and HL. However, such experiences, presumably, provide them with additional opportunities to learn about their bodies, which results in increased HL levels.

Interestingly, we found no significant differences in age and employment status between the two groups while they were experiencing PMS. One possible reason is that, although people may learn about health as they age, younger people seem to receive information through several channels, such as SNS. Women usually obtain health information, including information on issues related to pregnancy, STDs, and contraception, through the internet. Therefore, there were no significant differences in HL among age groups (i.e., under 29, 30-39, 40-49 age).

### 2. Association between HL and work performance

We found that women with high-HL were more likely to demonstrate health behaviors, and they had significantly lower presenteeism while experiencing PMS compared to those with low-HL. While our study is the first to observe an association between HL and work performance, a positive relationship between HL and health behaviors has been observed among a population of Japanese adults (including both men and women) and healthy Taiwanese women ^(19), (20)^. People who demonstrate appropriate health behaviors are more likely to be healthy ^(21)^. Moreover, there is evidence to suggest that healthy people are more likely to have better work performance ^(22)^. Thus, our findings suggest that, by using health behaviors, HL may enhance people’s health status and decrease presenteeism during PMS.

In contrast, we found no significant association between HL and absenteeism (i.e., women with high-HL were as likely to take time off from work as those with low-HL). Since we observed a strong association between HL and health behaviors, it seems more plausible to interpret this association as people with higher levels of HL not being reluctant to take sick leave when needed. Thus, while women with higher HL have better work performance when in the office, they do not spend more time in the office. A woman taking sick leave because of menstrual symptoms and PMS is a favorable, not an unfavorable, individual health behavior. On the other hand, absenteeism itself might reducing work productivity, so it may be important to practice health behaviors other than absenteeism to deal with menstrual abnormalities and PMS, which are serious enough to require sick leave.

### 3. Improving HL

It is essential to have opportunities for education on women's health in order to improve HL. In any educational institution, it is necessary to develop an environment to provide such education. In addition, it is necessary to provide the following opportunities to those who graduated from college: educational opportunities for women's health in the workplace and information sources, such as the internet and leaflets, with accurate information.

### 4. Strengths and limitations

This study’s strengths include its nationwide reach. The study participants’ ages and regional distributions were similar to Japan’s national demographic distribution. The study used a validated scale for HL and work performance.

However, the present study has several limitations. First, the causal relationships between caregiver burden and work productivity could not be determined because of the cross-sectional design. Therefore, longitudinal studies should be conducted. Second, this study was based on survey data, collected via self-report measures. In addition to self-report bias (such as negative affect), common method variance might have affected the results, suggesting that the true associations between variables might be weaker than those observed in this study. Recall bias might have also occurred in terms of presenteeism while experiencing PMS because we asked about past PMS and/or menstrual symptoms. Third, our data were collected via the internet; therefore, our findings’ generalizability may be challenged. The socioeconomic status and educational status of the average internet user may be greater than those of the general population ^(23)^. Thus, similar to typical internet studies, self-selection might be one of the present study’s limitations. Fourth, the web-based survey in the present study might be accessible by those who were unemployed. However, the registration information of participants, such as names, ages, and e-mail addresses, were checked regularly to prevent unauthorized access. Therefore, this influence was minimized. Finally, minimal consideration was given to unmeasured factors, such as work-related factors (i.e., healthcare worker or non-healthcare worker status, working hours, and shift working or non-shift working), job stressors (e.g., job demands and job control), and personal characteristics (e.g., smoking, alcohol intake, exercise, history of, and/or current illnesses, such as depression, physical disability, or other unknown factors). These potential confounders may have influenced the relationship between HL and work performance. Lastly, to the best of our knowledge, almost no previous studies have investigated the association between HL and work productivity. Therefore, we aimed, primarily, to investigate the association between the two. Hence, this study has not yet been able to investigate concrete mechanisms. Possible mechanisms between HL and work productivity are: 1) In health literacy studies, some researchers have suggested that an association between health literacy and health behavior^(20), (21)^; 2) On the other hand, health behaviors may have influenced work performance (i.e., positive health behaviors lead to preventive health measures and investments in health, which can improve and maintain the health status of workers and increase work performance) ^(3)^. Therefore, HL appears to lead to positive health behavior, which results in good work performance. However, more investigations are necessary, including the elucidation of the mechanisms.

### 5. Conclusions

This study aimed to examine the association between HL and work productivity in female Japanese workers. We also tested for associations between HL and health behaviors as a secondary outcome, which has also been reported in previous studies. The results indicated a significant positive association between HL level, work performance, and health behaviors.

## Article Information

### Conflicts of Interest

This research was supported by Bayer Yakuhin, Ltd. and docomo Healthcare, Inc. The sponsors had no control over the data analysis, data interpretation, or writing of this manuscript.

### Acknowledgement

We would like to express our sincere gratitude to Dr. Yuji Taketani (Chairperson, Artemis Women’s Hospital), who kindly provided us with informative and valuable advice throughout this study.

### Author Contributions

YI and KK designed the study and wrote the initial draft of the manuscript. KK and SS contributed to analysis and interpretation of data and assisted preparation of the manuscript. All authors have contributed to data collection and interpretation and critically reviewed the manuscript. All authors read and approved the final manuscript.

Yuko Imamura and Kazumi Kubota contributed equally to this work.

### Approval by Institutional Review Board (IRB)

The survey was conducted with the approval of the Ethics Review Committee of the Health Outcome Research Institute, Japan. The approval code is 2018-001.
